# The end of the line? A case of drug resistance to third-line antiretroviral therapy

**DOI:** 10.4102/sajhivmed.v17i1.454

**Published:** 2016-05-31

**Authors:** Theresa M. Rossouw, Sonia Hitchcock, Mariëtte Botes

**Affiliations:** 1 Department of Immunology, University of Pretoria, South Africa; 2 Department of Family Medicine, University of Pretoria, South Africa; 3 Kalafong Provincial Tertiary Hospital, South Africa; 4 Muelmed Hospital, Pretoria, South Africa

## Abstract

HIV drug resistance has been described in all antiretroviral drug classes and threatens the long-term success of HIV treatment. Here, we describe the first reported case of acquired resistance to the integrase strand transfer inhibitors in South Africa. This case illustrates the dilemma of treatment in the context of inadequate adherence and poor psychosocial support and highlights the potential risk of transmission of multidrug-resistant virus.

## Introduction

The development of drug resistance is one of the biggest concerns in the long-term management of HIV-infected patients. An increasing number of confirmed cases of drug resistance to second-line antiretroviral treatment (ART) has prompted the South African national treatment programme to introduce third-line treatment. This is, to the best of our knowledge, the first report of drug resistance to third-line therapy in the South African public sector. The Research Ethics Committee of the Faculty of Health Sciences of the University of Pretoria approved the case study and granted a waiver of consent.

### Patient presentation

**FIGURE 1:**
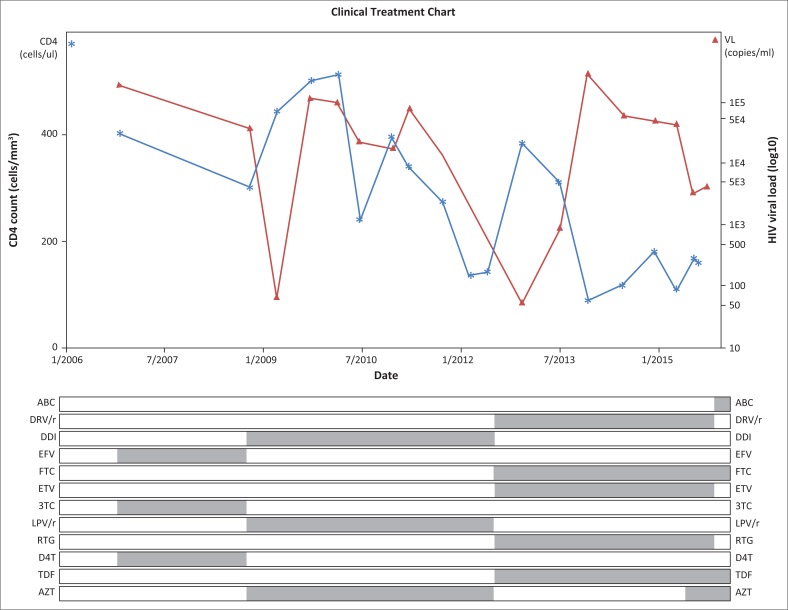
Antiretroviral treatment and monitoring data from 2006 to 2015.

The patient tested HIV-positive in 2006 at the age of 8 years. Her exposure to perinatal ART is unknown. She started her first ART regimen consisting of stavudine, lamivudine and efavirenz in the private sector in 2006 after completing a course of treatment for pulmonary tuberculosis. In 2007, she was transferred to the state sector. Here, she was changed to the standard second-line regimen in use at the time – zidovudine (AZT), didanosine and ritonavir-boosted lopinavir – because of virological treatment failure in October 2008. She initially responded well, but her viral load became detectable again in September 2009 and she was referred to an academic ART clinic in March 2010 because of virological failure. Her ART regimens, CD4 counts and HIV viral load results are shown in Figure 1.

### Management and outcome

At the time of the referral, the patient was 12 years old and living in Pretoria with her aunt. Her mother lived in Limpopo and was also using ART. The patient was aware of her HIV status and it was known to all her close adult family members. She reported poor adherence with her ART treatment because it was unpalatable.

She had three genotypic drug resistance tests (DRTs) over the next 2 years and the mutations evolved from low-level resistance to ritonavir-boosted lopinavir to extensive three-class resistance during this time (Table 1). She moved again to a private general practitioner in 2012 where she had another DRT performed at a private laboratory which confirmed the results of the second test. She was then changed to third-line therapy consisting of tenofovir (TDF), emtricitabine (FTC), ritonavir-boosted darunavir, raltegravir (RAL) and etravirine. The ARVs were delivered monthly from June 2012 to February 2015 by courier pharmacy and no parcels were returned. She attended all monitoring visits and initially had an excellent response to the third-line regimen.

**TABLE 1:** HIV genotypic drug resistance test results for 2011, 2012 and 2015.

Drug	2011		2012		2015	
			
	Mutations	Description of resistance	Mutations	Description of resistance	Mutations	Description of resistance
**NRTI mutations**	**None**	**-**	**D67N, T69AD, K70R, T215Y, K219Q**	**-**	**D67N, T69D, K70R, M184I/V/M**	-
Zidovudine	-	Susceptible	D67N, T69AD, K70R, T215Y, K219Q	High-level	D67N, K70R	Intermediate
Didanosine	-	Susceptible	D67N, T69AD, T215Y	Intermediate	D67N, T69D, M184I/V/M	Intermediate
Lamivudine	-	Susceptible	-	Susceptible	M184I/V/M	High-level
Stavudine	-	Susceptible	D67N, T69AD, K70R, T215Y, K219Q	High-level	D67N, T69D, K70R	Intermediate
Abacavir	-	Susceptible	D67N, T215Y	Low-level	D67N, M184I/V/M	Intermediate
Emtricitabine	-	Susceptible		Susceptible	M184I/V/M	High-level
Tenofovir	-	Susceptible	D67N, K70R, T215Y	Intermediate	D67N, K70R	Low-level
**NNRTI mutations**	**None**	**-**	**K103N**	**-**	**Y181C, E138Q/E, V179I/N/D/V**	**-**
Nevirapine	-	Susceptible	K103N	High-level	E138Q/E, V179I/N/D/V, Y181C	High-level
Efavirenz	-	Susceptible	K103N	Intermediate	V179I/N/D/V, Y181C	Intermediate
Etravirine	-	Susceptible	-	Susceptible	E138Q/E, V179I/N/D/V, Y181C	Intermediate
Rilpivirine	-	Susceptible	-	Susceptible	E138Q/E, V179I/N/D/V, Y181C	Intermediate
**PI mutations**^a^	**V82AV, (T74S)**	**-**	**M46I, I54V, L76V, V82A, (L10F)**	-	**M46I, L76V, V82A, (L10F, L24I, V32A, L33F, K43T, T74S)**	-
Saquinavir/r	V82AV, (T74S)	Potential low-level	I54V, V82A	Intermediate	V82A, (L24I, K43T)	Low-level
Indinavir/r	V82A	Intermediate	M46I, I54V, L76V, V82A, (L10F)	High-level	M46I, L76V, V82A, (L10F, L24I, K43T)	High-level
Nelfinavir	V82AV, (T74S)	Intermediate	M46I, I54V, V82A, (L10F)	High-level	M46I, V82A, (L10F, L24I, L33F, K43T, T74S)	High-level
Fosamprenavir/r	V82AV, (T74S)	Potential low-level	M46I, I54V, L76V, V82A, (L10F)	High-level	M46I, L76V, V82A, (L10F, L24I, L33F, K43T)	High-level
Lopinavir/r	V82A	Low-level	M46I, I54V, L76V, V82A, (L10F)	High-level	M46I, L76V, V82A, (L10F, L24I, L33F, K43T)	High-level
Atazanavir/r	V82AV, (T74S)	Low-level	M46I, I54V, V82A	Intermediate	M46I, V82A, (L24I, K43T)	Intermediate
Tipranavir/r	-	Susceptible	I54V	Potential low-level	(L33F, K43T)	Low-level
Darunavir/r	-	Susceptible	L76V, (L10F)	Low-level	L76V, (L10F, L33F, K43T)	Intermediate
**INSTI mutations**	**n/a**	**-**	**n/a**	-	**T97A, V151I, N155HINI, E157E/Q**	-
Raltegravir	-	-	-	-	T97A, E157E/Q, N155HINI	High-level
Elvitegravir	-	-	-	-	T97A, E157E/Q, N155HINI	High-level
Dolutegravir	-	-	-	-	T97A, E157E/Q, N155HINI	Low-level

NRTI, nucleoside/nucleotide reverse transcriptase inhibitors; NNRTI, non-nucleoside reverse transcriptase inhibitors; PI, protease inhibitors; INSTI, integrase strand transfer inhibitors.

a , Major mutations with minor mutations in brackets.

However, by 2013 the patient was not coping well and had developed urinary and faecal incontinence without a physiological cause. She had nearly been raped, had failed grade 9 and had become socially isolated. By December 2013, her viral load reflected the impact of the psychological problems on her adherence. Her mother passed away in 2015 and with that the patient lost her medical aid benefits and was referred back to the state sector. A fourth DRT (Table 1) showed that resistance to the non-nucleoside reverse transcriptase inhibitors and protease inhibitors had worsened and also now included resistance to the integrase strand transfer inhibitors (INSTIs).

Currently, neither the patient nor the family seem to have grasped the seriousness of her condition. Despite multiple adherence counselling sessions and even contracting with the family that her pill-taking will be supervised, there is still inadequate supervision. She has poor coping skills, has not entirely accepted her illness and is ambivalent about taking her ART. She is now on a holding regimen consisting of FTC, TDF, AZT and abacavir (ABC). A tropism assay predicted that CCR5-antagonists are likely to be effective.

## Discussion

This patient has come to the end of the line in terms of available ART. The virus displays intermediate to high-level resistance to all the available ARVs (either in the second or the third genotype) and even low-level resistance to the new INSTI, dolutegravir (DTG) and the new-generation protease inhibitor (PI), tipranavir. A potential new regimen should therefore preferably include an entry inhibitor (e.g. maraviroc) and tipranavir, together with TDF, FTC and DTG. The cost, however, is prohibitive and apart from TDF/FTC, none of the medications are available in the public sector at this time.

This case raises the question about the positioning of DTG in the treatment guidelines once it becomes available in South Africa. A considerable minority of patients develop cross-resistance to DTG after exposure to RAL and elvitegravir, while resistance has only rarely been reported in patients exposed to DTG as the first integrase inhibitor.^1,2^ This case also illustrates the complexity of the long-term management of HIV in the context of poor psychosocial support and suboptimal adherence. It raises concerns about the addition of drug classes in the face of ongoing non-adherence and highlights how management is complicated, and unnecessary costs incurred, when patients move between health sectors without an electronic patient record.

This case also raises the issue of potential transmission of extensively drug-resistant virus in a teenager who is bound to become sexually active in the foreseeable future. Transmitted multiclass (including INSTI) resistance has been reported in the United States,^3,4,5^ and clinicians have called for INSTI resistance testing for patients with an unexpectedly high number of pre-ART drug resistance mutations and for contacts of heavily treatment-experienced patients.^6^ This may become a reality in South Africa if efforts to prevent the emergence of resistance are not prioritised.

## Conclusion

This case of acquired multiclass resistance, including resistance to INSTIs, in a teenager in South Africa illustrates the complexity of the long-term management of HIV in contexts where inadequate adherence and poor psychosocial support abound and highlights the importance of the appropriate positioning of DTG in the national treatment programme.
